# Supplementary material for: Optimizing motor decision-making through competition with opponents
Source: Sci Rep. 2020 Jan 22;10:950. doi: 10.1038/s41598-019-56659-6 (PMC6976621; doi:10.1038/s41598-019-56659-6)
Supplement: Supplementary file 1 — Supplementary information. [file 41598_2019_56659_MOESM1_ESM.pdf]

Supplementary information

Optimizing motor decision-making through competition with opponents

Keiji Ota, Mamoru Tanae, Kotaro Ishii, and Ken Takiyama

Keiji Ota

Email: keiji.ota@nyu.edu

This PDF file includes:

Supplementary text

Supplementary Figures 1 to 9

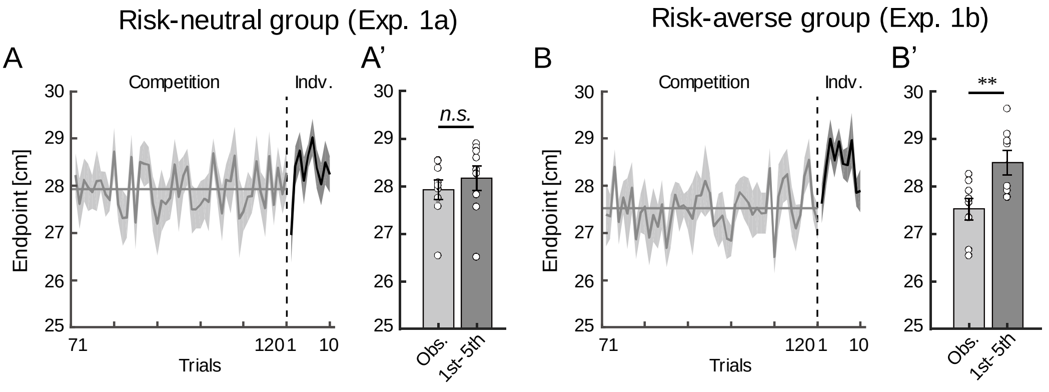

Supplementary Figure 1. Reversal of risk-sensitivity is not observed from the competitive task to the individual task.

(**A** and **B**) Time series of the reaching endpoint from the competitive task (competition) to the individual task (washout). Data is averaged across the subjects, and the shaded area denotes the standard error of the mean. The horizontal solid line indicates the observed mean endpoint in the competitive task for 50 trials (blocks 8–12) before the individual task (washout) started. (**A’** and **B’**) Obs. indicates the observed mean endpoint in the competitive task, and 1st–5th indicates the average endpoints across the first to fifth trials after the individual task (washout) started. ** represents $p < 0.01$ (paired t-test). Open circles denote the data for each subject. A significant decrease in the reaching endpoint was not observed when the competitive task was switched to the individual task.

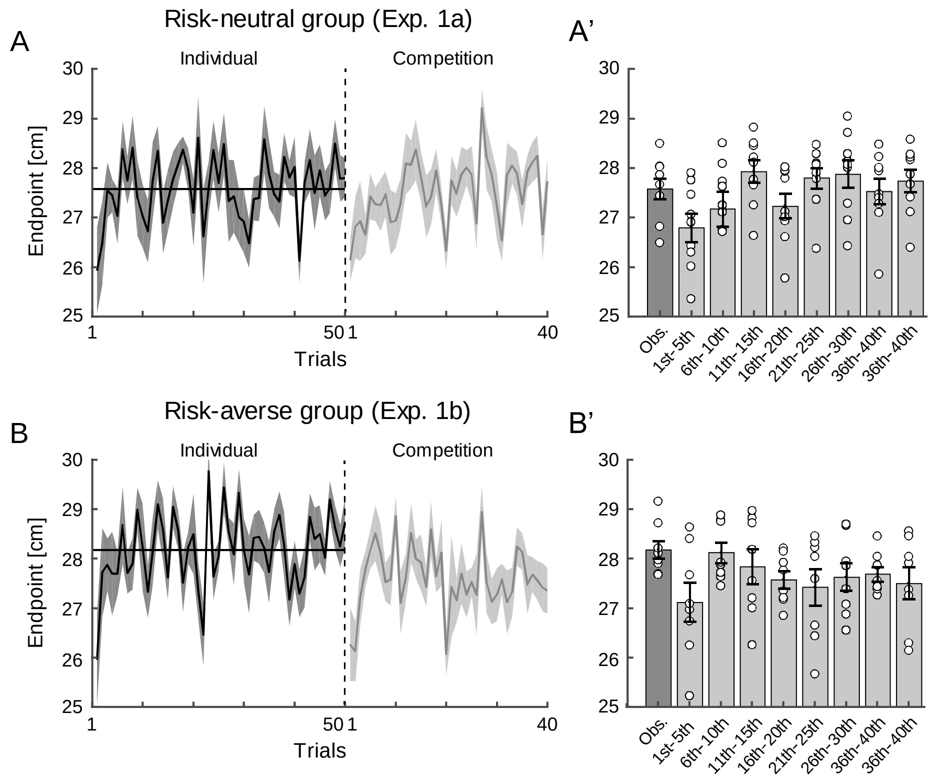

Supplementary Figure 2. Time-series of the reaching endpoint in the first 4 blocks of the competitive task.

(**A** and **B**) As supplementary material for Fig. 4, we show the time-series of the endpoint after the second block of the competitive task. (**A’** and **B’**) Obs. indicates the observed mean endpoint in the individual task, and light gray bars represent the averaged mean endpoint in each bin of 5 trials.

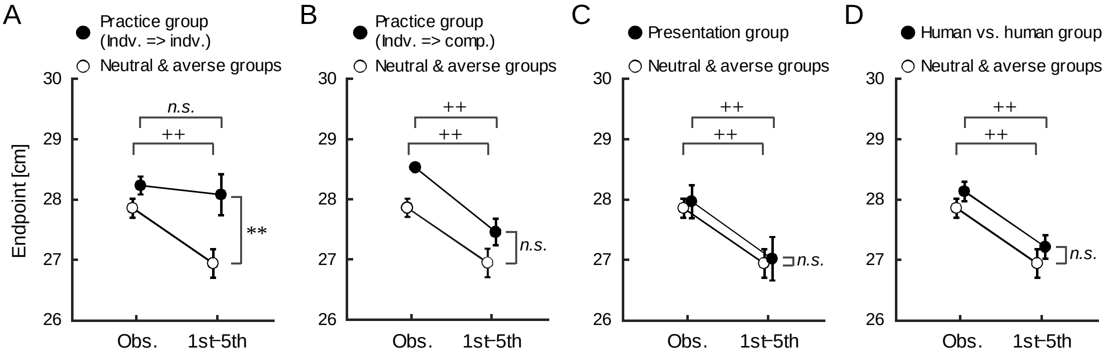

Supplementary Figure 3. Between-group comparison of reaching endpoints.

(**A**) Obs. indicates the observed mean endpoint in the individual task (baseline), and 1st–5th indicates the average endpoints across the first to fifth trials after the competitive task started in the risk-neutral and risk-averse groups (open circle) or when the individual task restarted in the practice group (filled circle). The filled circle (practice group, N = 10) is replotted from Fig. 4C'. For the open circle, we pooled the data in the risk-neutral and risk-averse groups (N = 17) because the opponents at the first block of the competitive task were the same between groups. The difference in the data point represents the difference in terms of whether the subjects competed with the computer opponent or not. Two-way mixed design ANOVA revealed a significant interaction between the experimental group (2: practice group or neutral and averse groups) and session (2: Obs. or 1st–5th) (F [1, 25] = 4.28, $p = 0.049$, $\eta^2 = 0.04$). The average endpoint across the first to fifth trials after the competitive task started was significantly lower than that after the individual task restarted ($p = 0.009$), suggesting that the competition with the computer opponent induced a shift in strategy. ** represents $p < 0.01$ between groups. ++ represents $p < 0.01$ from baseline. Error bar represents the standard error of the mean. (**B**) The filled circle (practice group) is replotted from Fig. 4D'. The open circle is the same as Supplementary Fig. 3A. Obs. in the practice group (filled circle) indicates the observed mean endpoint in the individual task for 50 trials before the competitive task started. The difference in the data point represents the difference in terms of whether the subjects practiced the individual task for a long period before the competition or not. Two-way mixed design ANOVA showed no significant interaction between the experimental group and session (F [1, 25] = 0.25, $p = 0.62$, $\eta^2 = 0.00$). There was no significant difference between the average endpoint after the competitive task started in the practice group and that in the neutral and averse groups ($p = 0.16$). (**C**) The filled circle (presentation group, N = 11) is replotted from Fig. 4E'. The difference in the data point represents the difference in terms of whether the subjects observed the performance of the computer

opponent before the competition or not. Again, two-way mixed design ANOVA showed no significant group × session interaction ($F [1, 26] = 0.01$, $p = 0.91$, $\eta^2 = 0.00$). There was no significant difference of the average endpoints after the competitive task started between groups ($p = 0.86$). (**D**) For the filled circle, we pooled two subjects' data ($N = 28$) in the human vs. human group to see the effect of competing with a human opponent on the decrease of the endpoint. The difference in the data point represents the difference in terms of whether the subjects competed with a human opponent or computer opponent. Similarly, two-way mixed design ANOVA showed no significant group × session interaction ($F [1, 43] = 0.001$, $p = 0.98$, $\eta^2 = 0.00$). The average endpoint after the competitive task in the human vs. human group was not significantly different from that in the neutral and averse groups ($p = 0.29$).

Taken together, these results suggest that a shift in strategy (a decrease in the endpoint) was shown in the very beginning of the competition and that it was not influenced by practicing a reaching movement, observing the opponent performance in advance, or competing with a human opponent.

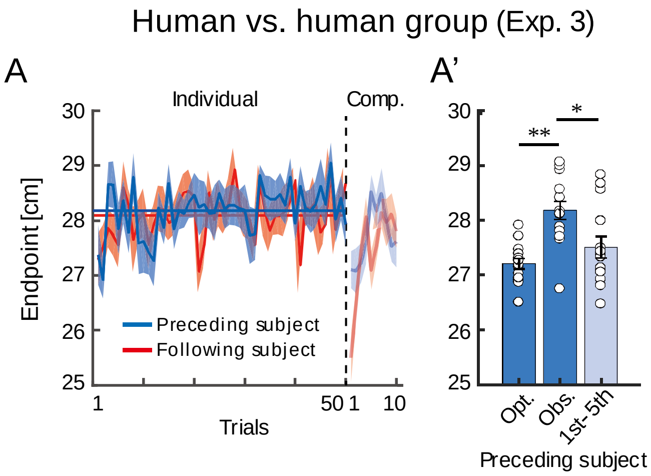

Supplementary Figure 4. Results for the preceding subjects in the human vs. human group. (**A**) Time series of the reaching endpoint in the individual and competitive tasks. Data is averaged across the subjects, and the shaded area denotes the standard error of the mean. Horizontal blue and red lines indicate the observed mean endpoint in the individual task (baseline) for the preceding subject (i.e., the subject who played first) and the following subject, respectively. (**A'**) Bar graphs show the averaged reaching endpoint across the preceding subjects. Open circles denote the data for each preceding subject. Similar to the results for the following subjects (Fig. 4F and F'), the preceding subjects showed a significant decrease in the reaching endpoint at the beginning of the competitive task.

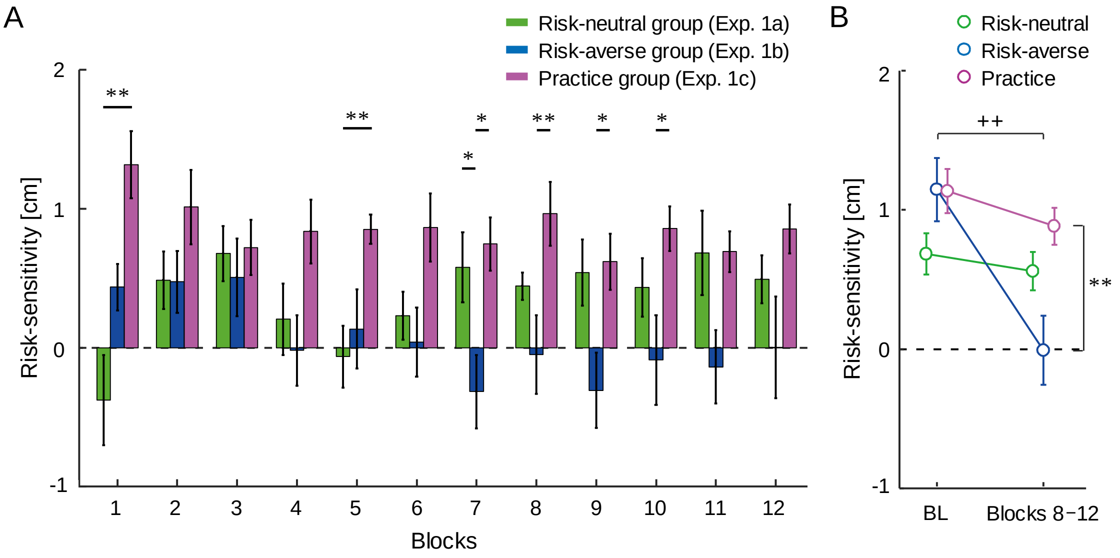

Supplementary Figure 5. Between-group comparison of risk-sensitivity values.

(**A**) In this panel, Figure 5 is replotted as a bar graph to clarify the group differences. Risk-sensitivity values, defined as the difference between the mean endpoint and optimal endpoint, are plotted for each block of the competitive task (neutral and averse groups) or the individual task (practice group). Two-way mixed design ANOVA revealed a significant group [3] × block [12] interaction ($F[22, 264] = 2.12$, $p = 0.002$, $\eta^2 = 0.09$). The risk-sensitivity values in the risk-averse group were significantly smaller than those in the practice group in blocks 7, 8, 9, and 10 ($ps < 0.05$, Bonferroni correction). (**B**) The risk-sensitivity values at the baseline (individual task) and for blocks 8th–12th of the competitive task (neutral and averse groups) or the individual task (practice group) are plotted. Two-way mixed design ANOVA revealed a significant group [3] × session [2] interaction ($F[2, 24] = 10.44$, $p = 0.001$, $\eta^2 = 0.12$). In blocks 8–12, the risk-sensitivity values in the risk-averse group were significantly smaller than those in the practice group ($p < 0.01$, Bonferroni correction). * indicates $p < 0.05$ and ** indicates $p < 0.01$ between groups. ++ indicates $p < 0.01$ from baseline.

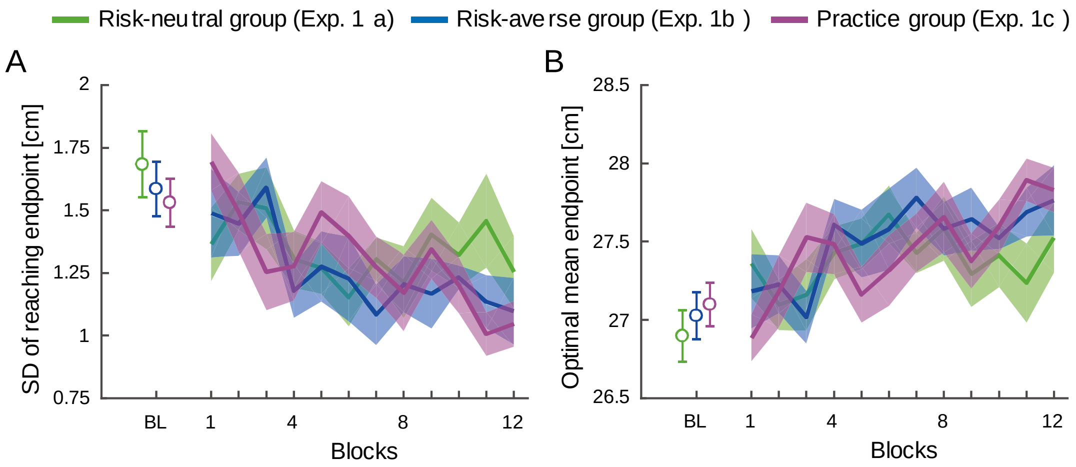

Supplementary Figure 6. No difference in terms of reaching accuracy or optimal mean end point among the three groups.

(**A**) Average standard deviation of the reaching endpoint across the subjects. (**B**) Given the SD of the endpoint, the optimal mean endpoint was calculated based on equation [3] in the main text. The shaded area denotes the standard error of the mean. Two-way mixed effects ANOVA showed no significant group × block interaction ($F [22, 264] = 1.14$, $p = 0.303$, $\eta^2 = 0.06$) and no main effect of group ($F [2, 24] = 0.36$, $p = 0.699$, $\eta^2 = 0.006$) for the standard deviation of the reaching endpoint. Similarly, there was no significant group × block interaction ($F [22, 264] = 1.15$, $p = 0.294$, $\eta^2 = 0.06$) and no main effect of group ($F [2, 24] = 0.35$, $p = 0.706$, $\eta^2 = 0.006$) for the optimal endpoint.

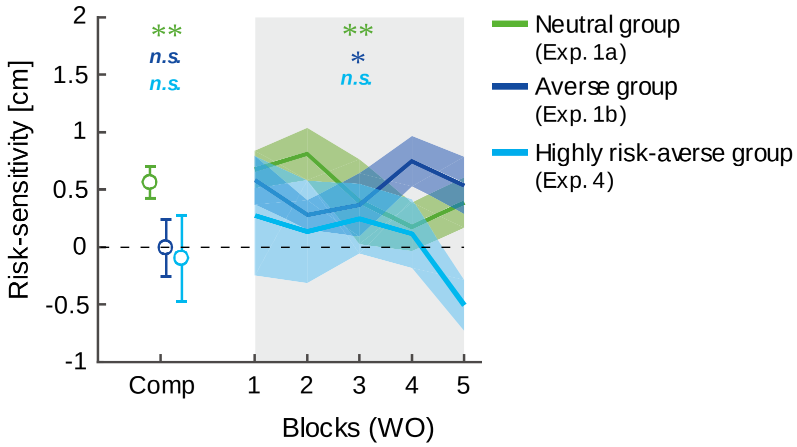

Supplementary Figure 7. Analysis of risk-sensitivity in the washout session.

Risk-sensitivity values in the risk-neutral (Exp. 1a), risk-averse (Exp. 1b), and highly risk-averse groups (Exp. 4) are plotted. Comp denotes the risk-sensitivity for the last 5 blocks (blocks 8–12) of the competitive task, whereas each block denotes the risk-sensitivity for each block of the washout session (individual task). Data is averaged across subjects, and the shaded area denotes the standard error of the mean. Positive values indicate a risk-seeking strategy. Double green asterisks denote $p < 0.01$ from the risk-neutral value (i.e. 0) for the risk-neutral and single blue asterisk denote $p < 0.05$ for the risk-averse group. At the washout, the optimal strategy was still retained in the highly risk-averse group even though there were no opponents. The risk-sensitivity in the 50 trials of the individual task (shaded gray area, from the 1th to 5th block of the washout session) was significantly different from 0 in the risk-neutral group (green line, two-tailed one-sample *t*-test from 0: t [8] = 5.00, p = 0.001, d = 2.50, mean difference = 0.61, 95% CI = [0.33, 0.89]) and the risk-averse group (blue line, two-tailed one-sample *t*-test from 0: t [7] = 3.24, p = 0.014, d = 1.74, mean difference = 0.56, 95% CI = [0.15, 0.96]). However, there was no significant difference in the highly risk-averse group (cyan line, two-tailed one-sample *t*-test from 0: t [5] = 0.66, p = 0.539, d = 0.42, mean difference = 0.19, 95% CI = [−0.55, 0.93]). The difference in this retention effect between the risk-averse and highly risk-averse groups might have originated from the difference in the experimental protocol, i.e., the latter subjects competed with the opponents who exhibited more highly risk-averse behavior and for a longer period.

Validation of model assumptions

The model in this study assumed that the subject's reaching variability σ^2 was the variability when their aim point was fixed at a particular point (see *Model assumptions*). One concern regarding this is that their aim point was not necessarily fixed while performing the individual or competitive task; in other words, the subjects might change their aim point from trial to trial. This variability in the aim point might increase the variability in the reaching movement σ^2 and influence the calculation of the optimal aim point. Here we investigated whether the variability in the aim point influenced the variability of reaching endpoint.

We recruited eight subjects (5 males, mean ± SD = 21.0 ± 2.2) and used two experimental tasks. The first task was the individual task. The subjects were instructed to maximize the total score; thus they might change their aim point in each trial. The other task was the training task. The subjects were instructed to reach the boundary line; thus the subjects' aim point was fixed at the boundary line in all trials. There were 7 experimental blocks (1 block = 50 trials). The individual task was performed in the 1st, 2nd, 4th, and 6th block, whereas the training task was performed in the 3rd, 5th, and 7th block. In the training task, we presented the boundary line at the same distance as the mean endpoint in the preceding individual task. The mean distance of the boundary line was 28.4 cm.

Supplementary Figure 8 illustrates the standard deviation of the reaching endpoint in a block-by-block manner. To compare the difference between two tasks, we conducted two-way (2 [individual and training tasks] × 3 [blocks]) repeated measures ANOVA. As a result, there was no main effect of the task (F [1, 7] = 3.42, p = 0.11, η^2 = 0.05) and no significant interaction (F [2, 14] = 0.68, p = 0.52, η^2 = 0.02). There were no significant differences between blocks 2 and 3 (p = 0.08, mean difference = 0.13, CI = [−0. 02, 0.28]), between blocks 4 and 5 (p = 0.44, mean difference = 0.06, CI = [−0.12, 0.24]), and between blocks 6 and 7 (p = 0.64, mean difference = 0.03, CI = [−0.10, 0.15]). Therefore, the difference in terms of the reaching variability was marginal (average SD of the reaching endpoint across the subjects: 1.14 cm in the individual task and 1.06 cm in the training task). These results suggest that the assumption of the fixed aim point would not jeopardize our argument.

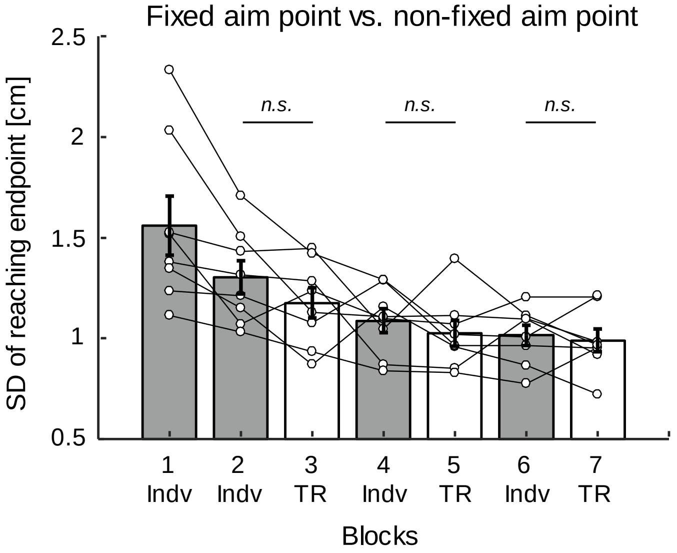

Supplementary Figure 8. Validation of model assumptions.

The standard deviation of the reaching endpoint is plotted as an index of the variability of reaching movement. The error bar denotes standard error of the mean. Each circle represents each subject.

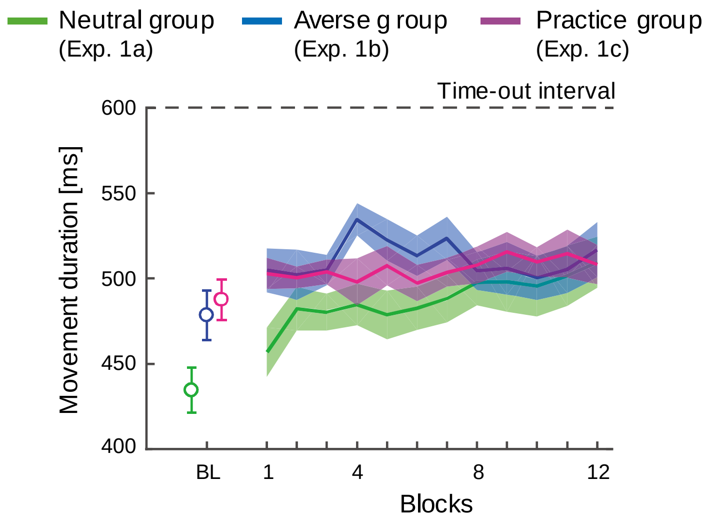

Supplementary Figure 9. Analysis of movement duration.

Average movement duration across the subjects. The shaded area denotes the standard error of the mean. The dashed line indicates the time-out interval (600 ms). Two-way mixed effects ANOVA showed no significant main effect of group (F [2, 24] = 1.30, p = 0.292, η^2 = 0.07) and main effect of block (F [4.10, 98.33] = 2.40, p = 0.053, η^2 = 0.03) but showed significant group × block interaction (F [8.19, 98.33] = 2.03, p = 0.049, η^2 = 0.04). There was a significant difference between the risk-neutral and risk-averse group at the 1st block (p = 0.037, Bonferroni correction), between the risk-neutral and practice group at the 1st block (p = 0.033), and between the risk-neutral and risk-averse group at the 4th block (p = 0.032).
